# Spatial Resolution of Microbial Community Members Within Cyanobacterial Mats From Temperate Mud Flats

**DOI:** 10.1002/ece3.72894

**Published:** 2026-01-07

**Authors:** Schyler A. Ellsworth, Madelina S. Marquez, Sophie J. McCoy

**Affiliations:** ^1^ Department of Biology University of North Carolina at Chapel Hill Chapel Hill North Carolina USA

**Keywords:** benthic cyanobacterial mats, community composition, nutrient cycling, vertical stratification

## Abstract

Benthic cyanobacterial mats (BCMs), known for their high cyanobacterial abundances, are distributed globally across diverse aquatic and semi‐aquatic habitats. The microbial composition of BCMs is determined by various environmental factors that regulate key chemical and physical conditions, leading to complex community structures that vary across space and time. Recently, temperate mats have emerged as model systems for studying community structure and nutrient cycling. Using 16S metabarcoding and stable isotope analyses, our study characterized the bacterial members present at a fine‐scale resolution across vertical layers of long‐standing BCMs from Shackleford Banks Island in North Carolina, USA. Spatially expansive BCMs were mainly composed of Proteobacteria, Bacteroidota, Planctomycetota, Desulfobacterota, Verrucomicrobiota, and Cyanobacteria. Mat communities did not differ along horizontal transects but differed in both the diversity and community composition between vertically stratified layers. Trends in organic matter, carbon, and nitrogen availability match changes in microbial composition and diversity across vertical strata. These findings provided the first molecular characterization of the full bacterial community from BCMs from North Carolina and provide insight into the potential biogeochemical processes within this system.

## Graphical abstract

We characterized and compared the bacterial community within intertidal and supratidal cyanobacterial mats from barrier islands in North Carolina across small horizontal and vertical spatial scales. We found that vertical layers within these stratified mats varied in their community composition and diversity together with nutrients and isotopic signatures and identified key taxa linked to these stratifications.

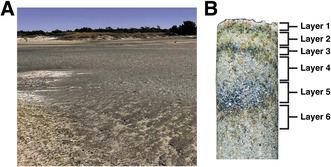

**Abbreviations**
δ^13^C: stable isotopic ratios of carbon
δ^15^N: stable isotopic ratios of nitrogen
ANCOM‐bc2: Analysis of Compositions of Microbiomes with Bias Correction 2
ASV: amplicon sequence variant
BCM: benthic cyanobacterial mat
CLR: center log ratio
TN: percent total nitrogen
TOC: percent total organic carbon
TOM: percent total organic matter

## 1 Introduction

Microbial mats are highly organized microbial assemblages that can span large contiguous areas (even up to a few thousand square meters; Valentine et al. 2016) and fulfill diverse ecosystem functions, including protecting shorelines from erosion (Hubas et al. 2011), storage of chemical energy sources and nutrients (Franks and Stolz 2009), and provision of sustenance and refuge for microfauna (Danovaro et al. 2024). The vertical layering of microbial mats produces many discrete microhabitats, generating steep gradients of metabolic and biogeochemical cycling (Chacón 2010). Benthic cyanobacterial mats (BCMs) are a type of microbial mat structured by high cyanobacterial abundances and hosting high phylogenetic and functional diversity of microbes (Cissell and McCoy 2021). They have a broad global distribution and are found in diverse aquatic and semi‐aquatic habitats, ranging from the tropics, documented for example in the Caribbean, Fiji, and Bali, to more subtropical and temperate regions such as the Mediterranean Sea, and Eastern United States, and extending into high latitude regions, such as Antarctica (Cissell et al. 2019; Ford et al. 2018; Fernandez‐Valiente et al. 2007; Cissell and McCoy 2024).

The global and local environment of a BCM strongly influences its composition due to its impact on the availability of key chemical resources (oxygen, pH, redox potential, and nutrients) as well as physical resources (light and temperature) (Prieto‐Barajas et al. 2018). Because of this, BCMs often exhibit similar vertical stratification due to universal responses to light and redox gradients approaching the sediment interface (Paerl and Pinckney 1996; Hoehler and Jørgensen 2013). Generally, BCM thickness can range from millimeters to centimeters, formed by diverse microbial communities that are structurally embedded in exopolysaccharides (Prieto‐Barajas et al. 2018). Although BCM structures are extremely variable, vertical zonation in temperate BCMs typically consists of a surface layer containing oxygenic phototrophs, a transition zone where both microaerophilic and anaerobic processes overlap, and deeper anoxic zones dominated by fermenters, methanogens, and sulfate‐reducing bacteria (Paerl and Pinckney 1996; Nicholson et al. 1987). Characterizing the bacterial community and how it shifts within BCMs is paramount to understanding how these zones are delineated and to deciphering potential functional boundaries between suites of microbes.

Facilitation and metabolic cross‐feeding, wherein zones are recognized by microbes capable of specific biochemical functions, form the primary organizational basis for stratification patterns (Powell and McCoy 2024). For example, phototrophic cyanobacteria in the uppermost layers generate oxygen and organic matter, which is consumed by heterotrophic bacteria in the microaerophilic zone, releasing carbon dioxide and byproducts such as acetate and hydrogen that diffuse further downwards to fuel anoxic sulfate‐reducing bacteria. The products of sulfate‐reducing bacteria (hydrogen sulfide) can then rise into light‐exposed zones, supporting anoxygenic phototrophs (e.g., purple sulfur bacteria) for photosynthesis (Prieto‐Barajas et al. 2018; Nicholson et al. 1987; Buckley et al. 2008). These processes are dynamic and complex, with carbon, sulfur, oxygen, and nitrogen cycling processes all interconnected in both time and space (reviewed in Powell and McCoy 2024). Understanding the fine‐scale structure of these communities and their involvement in biogeochemical cycles will provide the background needed to understand how BCMs form and persist.

BCMs exhibit both spatial and functional dynamics that underscore their complexity and importance in driving critical cycling processes across multiple ecosystems. While variation within microbial mats has been documented within stratified mats (Gomes et al. 2020; Armitage et al. 2012; Boidi et al. 2022) across large spatial scales (Huang et al. 2020; Farias et al. 2017; Bolhuis and Stal 2011; Wang et al. 2023; Stuij et al. 2023), there has yet to be a study of the fine‐scale variability of the perennial marine BCMs found on barrier islands on the temperate Atlantic coast of the United States, which have been studied intensively for their role in critical coastal biogeochemical processes (Paerl et al. 1993, 1991; Bebout et al. 1987; Bautista and Paerl 1985). BCMs from Shackleford Banks Island in North Carolina, USA, have received little characterization of the bacterial members present besides the cyanobacteria responsible for mat formation and nitrogen fixation (Paerl et al. 1991, 1993; Bebout et al. 1987). Building on this foundation, the present study will add to existing datasets of community members previously identified through microscopy via metabarcoding in combination with nutrient and isotopic analyses. Through investigating the fine‐scale community structure of temperate BCMs and the available nutrient and isotopic signatures, we add to the growing literature on BCM structure and function.

## 2 Materials and Methods

### 2.1 Study Site Description

Water bodies in Carteret County, NC (located in the Central North Carolina coast, near Beaufort County) surround the island of Shackleford Banks (Latitude: 34°41′12.8″ N, Longitude: −76°38′58.9″ W), which is a part of the Cape Lookout National Seashore. This island, about 9 miles long, is a part of the southern barrier islands of North Carolina and is known for its rich ecosystem diversity despite its narrow, windswept geography. The island consists of various marine, estuarine, and terrestrial ecosystems, such as beach and dune habitats, maritime grasslands and shrub thickets, salt marshes and intertidal flats, and shallow estuarine waters that each host specialized communities (Paerl et al. 1993).

**FIGURE 1:**
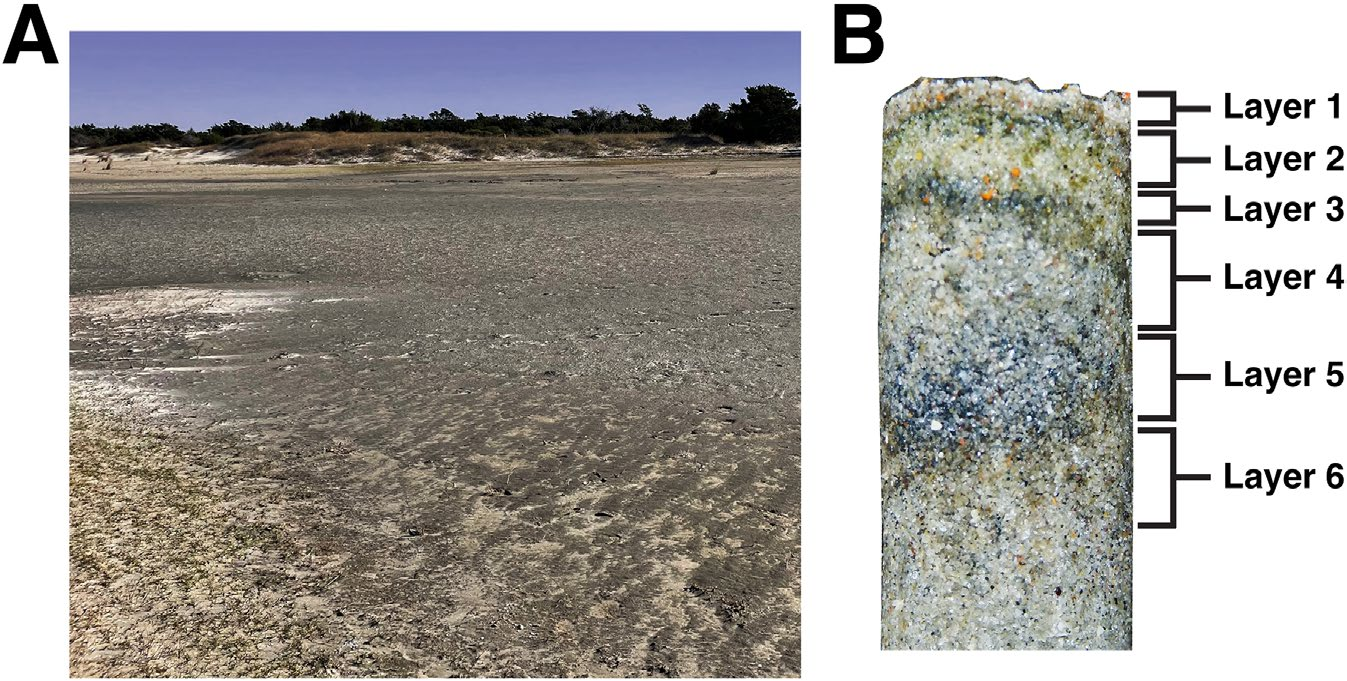
(A) Cyanobacterial mats found on the island of Shackleford Banks in Carteret County, North Carolina, USA. (B) Visual representation of vertical layers found within the cyanobacterial mats. Six vertical layers were separated based on the color and consistency.

BCMs are located within and surrounding a seasonally fluctuating saline‐hypersaline lagoon that is periodically inundated during extreme tidal or storm events, on the western tip of Shackleford Island (Figure 1). This area supports a year‐round cyanobacterial mat community due to its buffer from wave action (Bebout et al. 1987). Although not submerged on a regular tidal schedule, BCMs on Shackleford Island maintain daily moisture levels, suspected to be sustained by underlying groundwater seepage. Natural nutrient inputs within this area may be attributed to the proximity to Newport River, which flows into Back Sound, the body of water that separates the island from the mainland. Along with these natural inputs, several wastewater treatment plants in the vicinity may contribute to effluent that flows into estuarine systems surrounding Shackleford Island (Humphrey Jr 2009).

### 2.2 Sample Collection

Mats were sampled in triplicate during February 2024 from a continuous cyanobacterial mat bordering a sandy dune, with replicate samples collected using a small corer or modified syringe 5 m apart along the side of the mat closest to the sand dune. Biomass was immediately stored in DNA/RNA Shield (Zymo Research). Two different sampling types were taken at each replicated location on the mat. The first was sampled five times by a small corer (~5 mm diameter × 50 mm deep), starting at the edge of the mat and moving toward the middle of the mat at 0 cm (Core 1), 30 cm (Core 2), 70 cm (Core 3), 110 cm (Core 4), and 150 cm (Core 5) away from the edge. This sampling scheme aimed to determine whether the bacterial composition of the mat changed horizontally approaching the mat edge. The second sample type was a core taken by a modified 20 mL syringe (19 mm in diameter) with the bottom edge removed to allow the plunger to move all the way through the syringe, generating a core 70 mm in length. This corer was used to sample the center of a mat (150 cm away from the sandy edge) and split vertically into layers (Figure 1). Layers were determined by color and consistency. Layers were collected from 0 to 2.5 mm (Layer 1), 2.5 to 7.5 mm (Layer 2), 7.5 to 10 mm (Layer 3), 10 to 20 mm (Layer 4), 20 to 27 mm (Layer 5), and 27 to 35 mm (Layer 6). Two cores were collected with each replicate, with the layers from one core preserved in DNA/RNA Shield and duplicate layers collected in plastic bags then dried for isotopic analyses.

### 2.3 DNA Extraction and Sequencing

DNA was extracted from approximately 200 mg of each preserved core using the Omega E.Z.N.A. Soil DNA Kit following the manufacturer's recommendations. Extracted DNA was quantified using the Qubit dsDNA HS Quantification (Invitrogen). 3 μL of DNA was then used to target the 16S V4 region using universal bacterial barcodes 515F (5′‐GTGYCAGCMGCCGCGGTAA‐3′; Parada et al. 2016) and 806R (5′‐GGACTACNVGGGTWTCTAAT‐3′; Apprill et al. 2015) with added indices to allow for multiplexing of samples compatible with Illumina. The initial PCR amplification used 17 μL of a master mix of 0.1 μL of hot start Ex Taq DNA polymerase (Takara Bio), 1.6 μL of 2.5 mM dNTPs, 2 μL of 10× Ex Taq buffer, 1 μL of 2 μM of forward primer, 1 μL of 2 μM of reverse primer, and 11.4 μL of H_2_O per sample. For the amplification of the 16s fragment, we used an initial temperature of 95°C for 3 min followed by amplification cycles of 95°C for 1 min, 58°C for 2 min, and 72°C for 2 min. Samples were then left at 72°C for 10 min before storing at 4°C. Samples were then visualized on a gel to ensure amplification and cleaned with the GeneJET PCR Purification Kit (Thermo Scientific). To attach the Illumina barcodes, 3 μL from each sample was amplified with 3 μL of 1 μM dual indices, 0.1 μL of Ex Taq enzyme, 1.6 μL of 2.5 mM dNTPs, and 10.4 μL of H_2_O with an initial incubation for 3 min at 95°C followed by five cycles of 95°C for 1 min, 58°C for 1 min, and 72°C for 1 min. Samples were then left for 7 min at 72°C before being visualized on a gel to ensure amplification and attachment of the barcodes and quantified with the Qubit. All PCR amplifications contained negative controls to account for potential contamination. Samples and controls were then pooled with equal concentrations and cleaned using the GeneJET PCR Purification Kit. Pooled samples were then gel extracted using a 2% agarose gel loaded with 20 μL of the pooled sample. Samples were visualized using SYBR green on a blue light to extract the target band of approximately 500 base pairs. Gels were then cleaned with the GeneJET PCR Purification Kit and quantified on the Qubit before being sent to the University of North Carolina School of Medicine High Throughput Sequencing Facility. Samples were sequenced with paired‐end 250 base pairs reads on an Illumina MiSeq Platform with a spike‐in of 10% PhiX.

Raw reads were demultiplexed at the High Throughput Sequencing Facility. Adaptor sequences were trimmed from initial reads using Trim Galore (version 0.6.10; Krueger 2015) with quality controls of a minimum quality score of 20 and reads were greater than 100 base pairs. Primers were removed with Cutadapt (version 5.0; Martin 2011) using the forward and reverse primers and their complements. Reads were then checked to ensure that no primers were present by importing the data into R using Biostrings (version 2.70.3; DOI: 10.18129/B9.bioc.Biostrings) in R (version 4.3.2) and data was imported into the DADA2 pipeline (version 1.30.0; Callahan et al. 2016). Reads were subsequently filtered in DADA2 to remove PhiX reads and trim the sequences to 200 base pairs. Paired end reads were then merged, and an amplicon sequence variant (ASV) table was generated. Chimeric sequences were then removed from the merged reads to produce the final sequence variants for further analysis. One replicate from Core 2 was removed due to the low number of reads obtained from sequencing (394 reads). For the rest of the samples, between 46% and 77% of reads were kept and used for further analyses following filtering, merging, and chimera checks leaving between 37,746 and 80,944 reads for each sample.

### 2.4 Metabarcoding Analysis

Taxonomic classification was then assigned with the assignTaxonomy function in DADA2 using the Silva reference database (Version 138.1; Yilmaz et al. 2014; Quast et al. 2013). From the 35 samples, 10,015 ASVs were identified. After classification, the data were imported into Phyloseq (version 1.46.0; McMurdie and Holmes 2013), and any ASVs present in the negative control were removed from further analyses. Amplicon sequence variants were further trimmed to exclude mitochondrial, chloroplastic, and archaeal variants. Samples were then split into the horizontal variation experiment (Cores 1–5) and the vertical variation experiment (Layers 1–6 and Sand) for further analyses. To assess the diversity of bacteria found within the mats, we tested for differences in the richness and evenness present within the community using an analysis of variance model within the stats package in R. To test for normality and homogeneity of variance, we used the simulateResiduals function from the DHARMa package in R (version 0.4.7; Hartig 2016). After calculating alpha diversity metrics, underrepresented variants were trimmed using the MCMC.OTU package (version 1.0.10; Matz 2016). Variants were removed from the dataset if their sum of counts was below 0.001 of the fraction of the total counts and if they were present in less than 2% of the samples. This trimmed dataset resulted in 1683 ASVs and was used for the rest of the analyses.

Sample counts were then center log ratio (CLR) transformed for statistical analyses according to best practices for microbiome datasets (Gloor et al. 2017). Beta diversity was calculated using the vegan package (version 2.7‐1; Oksanen et al. 2007) and Euclidean distances between the CLR data. To test for differences in beta diversity, we used the adonis2 function in R. We tested for differences in beta dispersion using the betadisper function in vegan with bias adjust corrections followed by a permutation test.

A differential abundance analysis was performed on both the horizontal and vertical variation experiments using Analysis of Compositions of Microbiomes with Bias Correction 2 (ANCOM‐bc2, version 2.4.0; Lin and Peddada 2024). Raw sample counts were used in ANCOM‐bc2 as this package does not require the CLR transformed dataset. ANCOM‐bc2 was used at all taxonomic ranks using a global model with distance from the center of the mat and layers being used as categorical variables for the group predictor and fixed formula. Box plots of differentially abundant taxa and stacked bar plots were generated using ggplot2 (version 3.5.2; Wickham et al. 2016).

### 2.5 Nutrient & Isotopic Analysis

Frozen BCM biomass samples were placed in labeled foil boats and dried in the oven at 70°C for 48 h. Once dried, samples were homogenized using a mortar and pestle. Approximately 0.75 mL of homogenized sample was separated into two 1 mL portions, for carbon (C) or nitrogen (N) analyses, respectively. Samples for C measurement were acid washed to remove any inorganic carbon from the samples, such as carbonates. Acid washing was completed by adding 1 mL of 10% HCl to each sample tube and then inverting the tube. Once bubbling of CO_2_ was completed, excess HCl was removed by washing samples with ultrapure water, vortexing, and centrifuging to remove excess water. This rinsing step was repeated a second time to ensure all HCl was removed from the samples. Samples were then placed in the oven at 60°C for 24–48 h until completely dried. Once dried, samples for C and N measurement were weighed using an analytical balance (50–75 mg) and then encased within small tin capsules, utilizing a small scapula to move the sample and forceps to fold the sample closed within the tin capsule. Nutrient and isotopic analyses were conducted at the Cornell Isotope Laboratory using a Thermo Delta V isotope ratio mass spectrometer interfaced to a NC2500 elemental analyzer. The dataset returned contained isotopic (δ^13^C and δ^15^N) and elemental results (% total organic carbon and % total nitrogen).

We employed the Loss on Ignition (LOI) technique to measure the total percent organic matter within each sample. The remaining biomass of each sample after removing 2 mL for C and N analyses (described above) was placed into pre‐weighed foil boats and their precombustion weights were recorded (dried homogenized sample + foil boat). These samples were then placed within a combustion furnace at 500°C for 8 h. Post combustion, the samples were re‐weighed and subtracted from the pre‐combustion weights to calculate total percent organic matter within each sample.

Nutrient and isotope data collected for each sample included percent total nitrogen (TN), percent total organic carbon (TOC), percent total organic matter (TOM), and the stable isotopic ratios of carbon (δ^13^C) and nitrogen (δ^15^N). All nutrient & isotopic statistical analyses were completed in RStudio (version 4.2.1). Individual one‐way ANOVAs and pairwise comparisons were run for each response variable (TN, TOC, TOM, δ^13^C, δ^15^N). The assumptions of each ANOVA were confirmed: QQ plots and Shapiro–Wilk Tests for Normality and Levene‐Test for Homogeneity of Variance. If the ANOVA passed all the assumptions and was significant, then pairwise comparisons were performed using Tukey Multiple Comparisons of Means Test at the 95% family‐wise confidence level. If the assumptions of the ANOVA did not pass, then we used a nonparametric Kruskal–Wallis Rank Sum Test in conjunction with a Dunn's Test for pairwise comparisons (*p*‐values were adjusted for multiple comparisons with the Benjamini‐Hochberg method), mirroring other studies investigating nutrient analyses completed in coastal sediments, as well as looking at soil depth in a terrestrial environment (Haviland et al. 2022; Albert et al. 2021; Lorenz et al. 2020).

## 3 Results

### 3.1 Vertical Variation Experiment

**FIGURE 2:**
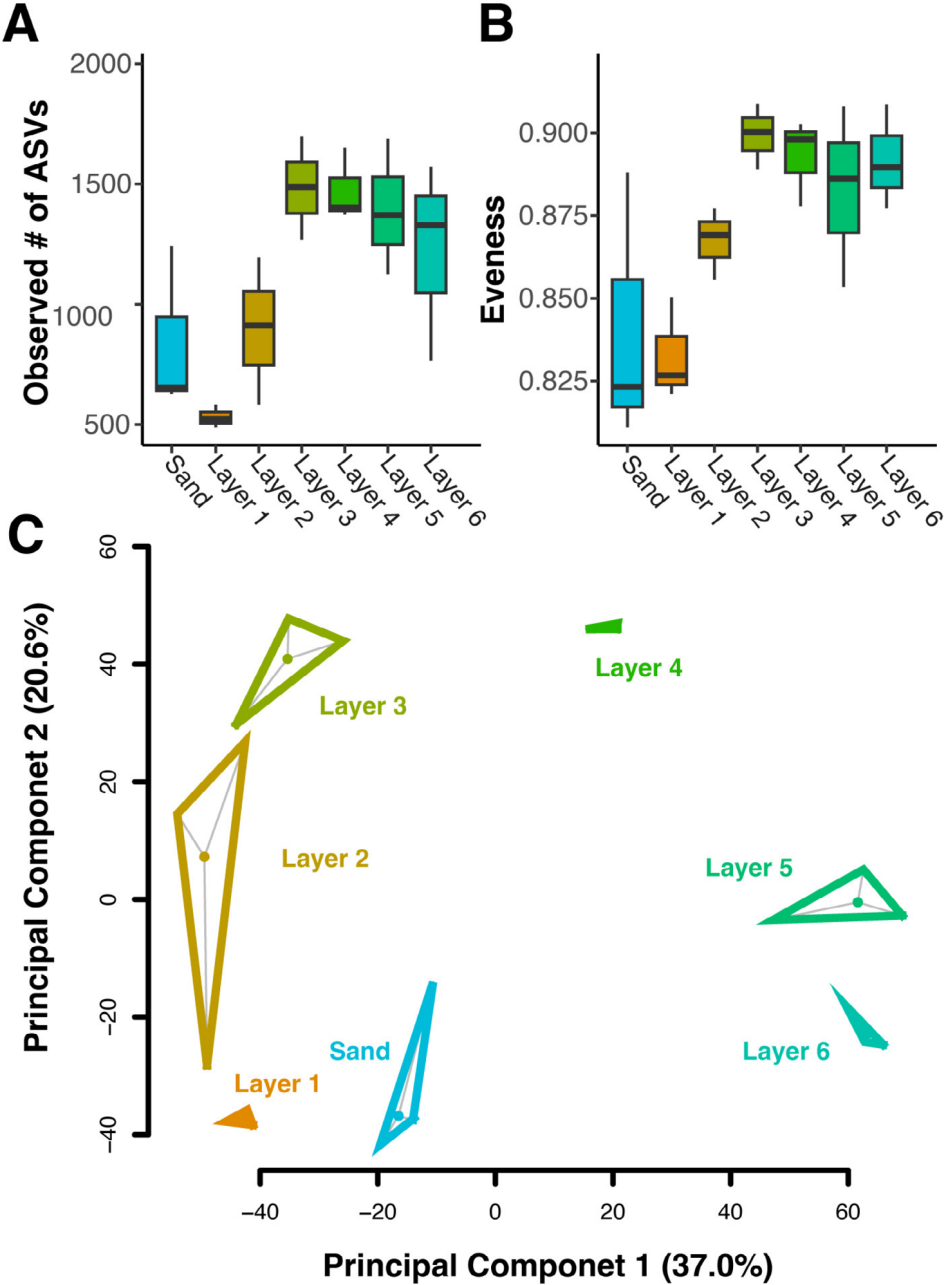
The number of species observed (A) and evenness (B) were both significantly different (*F*_6,14_ = 5.338, *p* = 0.00468 and *F*_6,14_ = 4.415 *p* = 0.0104, respectively). Beta dispersion (C) was not significantly different between layers (*F*_6,14_ = 1.0243, *p* = 0.4584), however beta diversity was significantly different (*F*_6,14_ = 5.5963, *p* < 0.001) as indicated by the difference between centroids.

**FIGURE 3:**
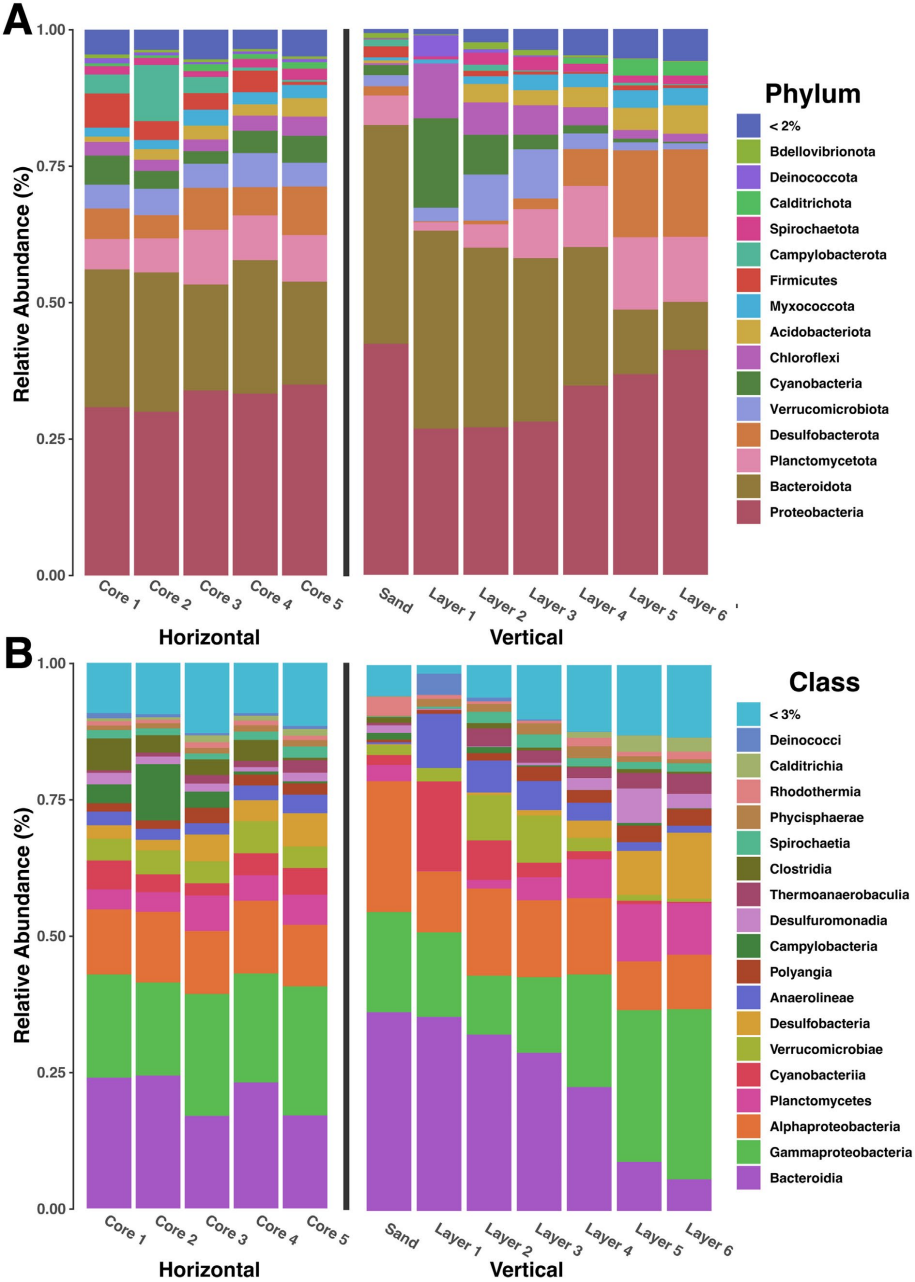
Relative abundance plots shown at the phylum (A) and class (B) taxonomic levels. The cores are shown on the left of the divide while the layers are shown on the right side of the divide. The phylum bar plot was limited to taxa that were above at least 2% in one of the samples. The class bar plot was limited to taxa that were at least 3% in one sample to limit the number of taxa being displayed.

**FIGURE 4:**
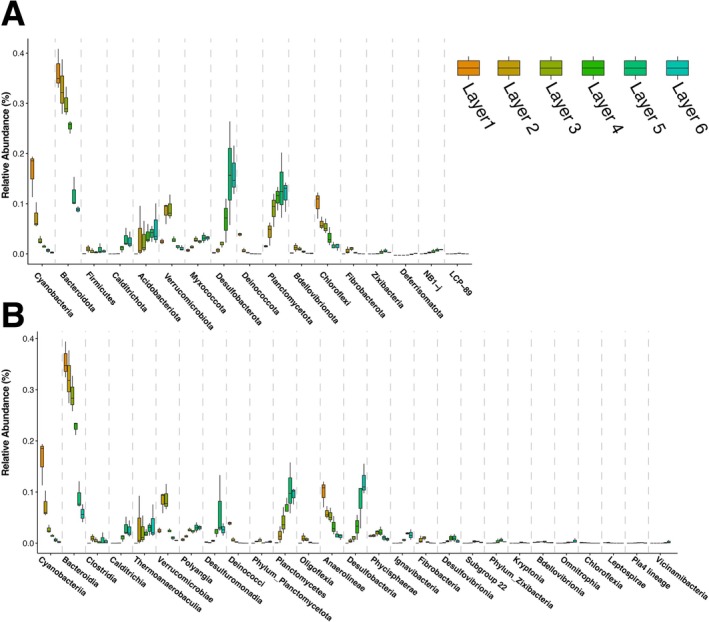
Box plots of the relative abundance of the phyla (A) and classes (B) that are differentially abundant between the layers. The *x*‐axis is organized by the taxa that were the most significantly different between the layers. The box plots within each taxon show the abundance of that taxon for each layer to highlight the trends of how taxon abundance differs between layers.

To identify potentially differences among the vertical stratifications found within cyanobacterial mats, we tested for differences in alpha and beta diversity and identified differentially abundant taxa between layers. We found that the number of observed species (*F*_6,14_ = 5.338, *p* = 0.0047, Figure 2) and the evenness (*F*_6,14_ = 4.415, *p* = 0.0104, Figure 2) differed significantly across layers. Samples collected from nearby sand and the first two layers drove the differences in the number of observed species and evenness (Figure 2). Beta dispersion between layers was not significantly different (*F*_6,14_ = 1.0243, *p* = 0.4584). However, beta diversity was significantly different (*F*_6,14_ = 5.5963, *p* < 0.001), indicating a difference between the composition of the communities between layers including sand. The most abundant phyla across the sand and layers were Proteobacteria, Bacteroidota, Planctomycetota, Desulfobacterota, Cyanobacteria, Chloroflexi, and Verrucomicrobiota (Figure 3). Seventeen phyla were significantly different in abundance across layers excluding sand (*p* < 0.001 for all phyla after Bonferroni correction): Cyanobacteria, Bacteroidota, Firmicutes, Calditrichota, Acidobacteriota, Verrucomirobiota, Myxococcota, Desulfobacterota, Deinococcota, Planctomycetota, Bdellovibrionota, Chloroflexi, Fibrobacterota, Zixibacteria, Deferrisomatota, NB1‐J, and LCP‐89 (Figure 4). The most abundant classes across layers were Bacteroidia, Gammaproteobacteria, Alphaproteobacteria, Planctomycetes, Cyanobacteriia, Anaerolinea, Verrucomicrobiae, and Desulfobacteria (Figure 3). Twenty seven classes were significantly different in abundance (*p* < 0.001 for all classes after Bonferroni correction), which were Cyanobacteriia, Bacteroidia, Clostridia, Calditrichia, Thermoanaerobaculia, Verrucomicrobiae, Polyangia, Desulfuromonadia, Deinococci, unidentified classes with the phylum Planctomycetota, Planctomycetes, Oligoflexia, Anaerolineae, Desulfobacteria, Phycisphaerae, Ignavibacteria, Fibrobacteria, Desulfovibrionia, uncultured class Subgroup 22 within the phylum Acidobacteriota, unidentified classes within the phylum Zixibacteria, Kryptonia, Bdellovibrionia, Omnitrophia, Chloroflexia Leptospirae, uncultured class Pla4 lineage within Planctomycetota, and Vicinamibacteria (Figure 4). Of these classes, Bdellovibrionia, Calditrichia, Chloroflexia, Clostridia, Cyanobacteriia, Deinococci, Desulfobacteria, Desulfovibrionia, Desulfuromonadia, Fibrobacteria, Ignavibacteria, Kryptonia, Oligoflexia, Omnitrophia, Phycisphaerae, unidentified classes with the phylum Planctomycetota, unidentified classes with the phylum Zixibacteria, Pla4, Planctomycetes, Polyangia, Subgroup 22, Thermoanaerobaculia, Verrucomicrobiae, and Vicinamibacteria were less than 2.5% in any of the layers.

### 3.2 Horizonal Variation Experiment

**FIGURE 5:**
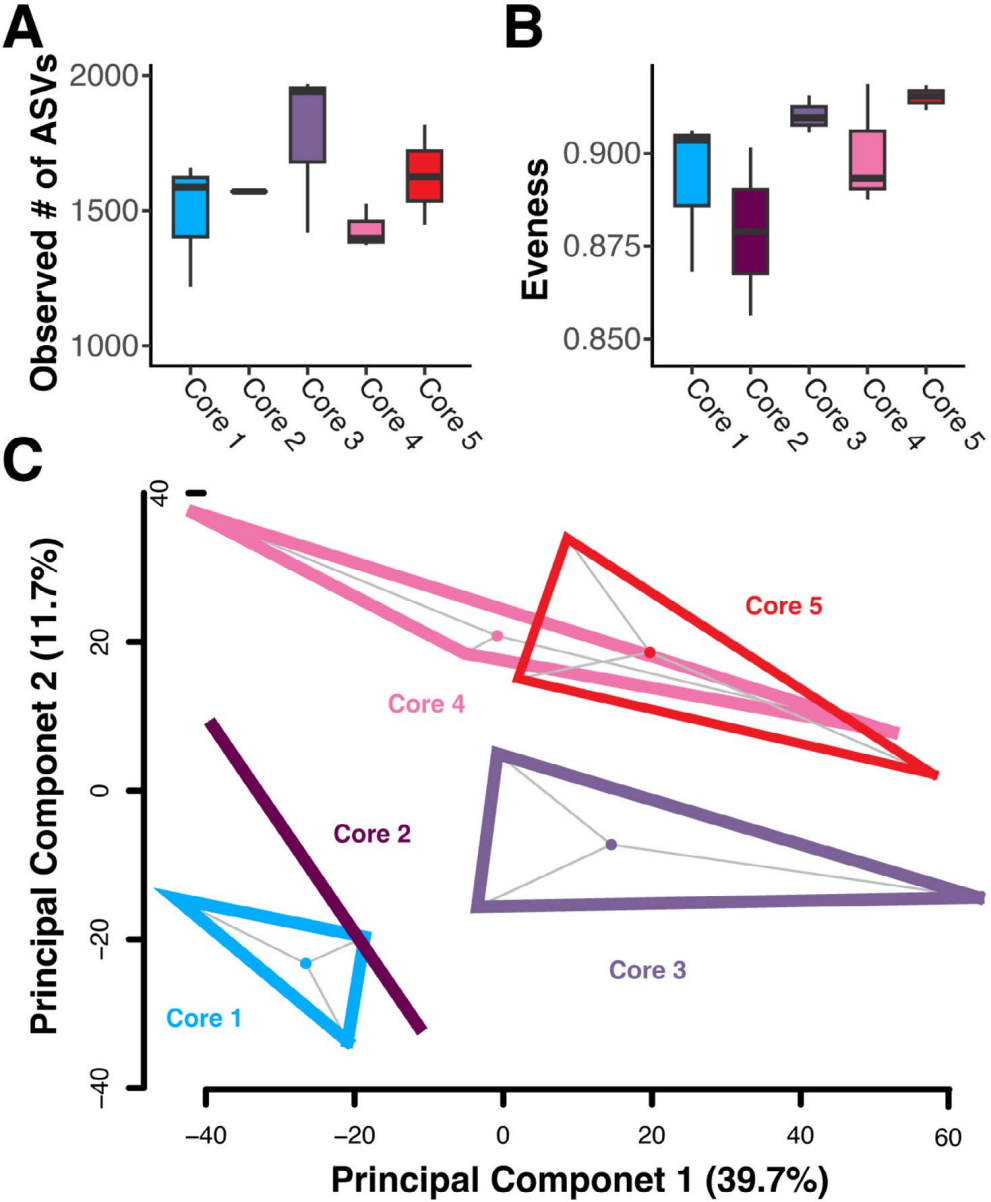
Alpha and beta diversity for the cores. The number of species observed (A), evenness (B), and beta diversity (C) were not significantly different across cores (*F*_4,9_ = 1.256, *p* = 0.355 for observed, *F*_4,9_ = 1.881, *p* = 0.211 for evenness, and *F*_4,9_ = 1.22, *p* = 0.1709 for beta diversity).

We investigated the variation found along a small‐scale horizontal gradient within a cyanobacterial mat by testing for differences in alpha and beta diversity and through identifying differentially abundant taxa between cores. We found no significant difference between species richness and the evenness between cores (*F*_4,9_ = 1.256, *p* = 0.355 and *F*_4,9_ = 1.881, *p* = 0.211, respectively, Figure 5). We also found no significant difference in beta diversity (*F*_4,9_ = 1.22, *p* = 0.1709) or beta dispersion (*F*_4,9_ = 0.4401, *p* = 0.7692, Figure 5). The most abundant phyla present across cores were Proteobacteria, Bacteroidota, Planctomycetota, Desulfobacterota, Verrucomicrobiota, and Cyanobacteria (Figure 3). Only Patescibacteria was significantly different between cores in the differential abundance analysis (*p* < 0.001). However, Patescibacteria was less than 0.3% of all phyla in any of the core samples. The most abundant classes were Bacteroidia, Gammaproteobacteria, Alphaproteobacteria, Planctomycetes, Cyanobacteriia, Verrucomicrobiae, Desulfobacteria, and Campylobacteria. Two classes significantly differed across cores, Gracilibacteria (*p* = 0.002) and uncultured class BD7‐11 within the phylum Planctomycetota (*p* = 0.006). However, Gracilibacteria and BD7‐11 were less than 0.5% and less than 0.2% of all classes in any of the core samples, respectively.

### 3.3 Results: Nutrient & Isotopic Analyses

**FIGURE 6:**
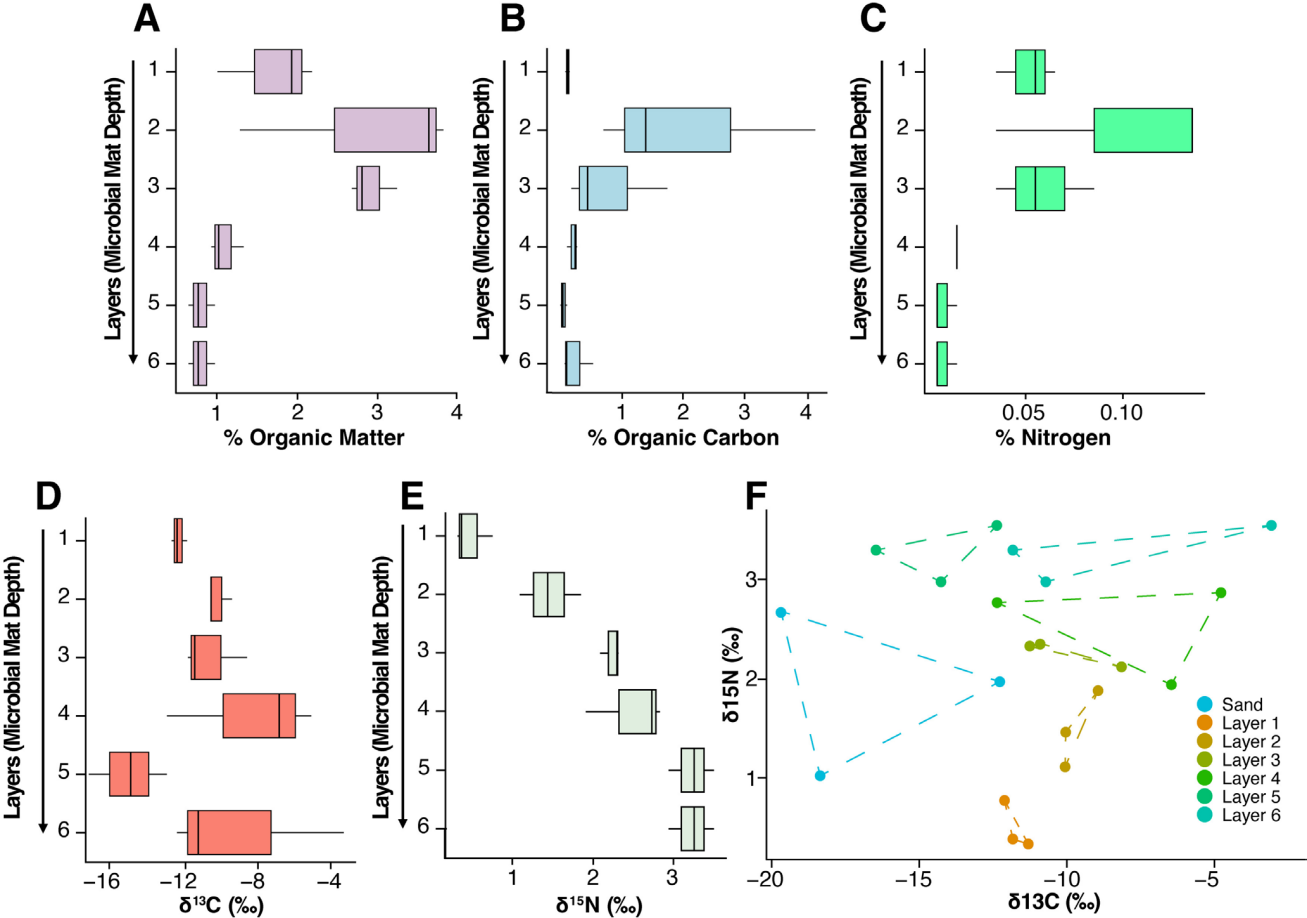
Boxplots of (A) percent total organic matter, (B) percent total organic carbon, (C) percent total nitrogen, (D) isotopic δ^15^N, and (E) isotopic δ^13^C concentrations throughout different depth‐associated layers of a microbial mat. (F) A scatter plot of both stable isotopic ratios across microbial mat samples. Each point represents a depth‐associated layer (Layer 1 = surface, Layer 6 = deepest). Sand controls were collected around a meter away from the microbial mats.

To determine if differences in community structure and diversity between the strata of BCMs were associated with organic matter or nutrient availability, we compared bulk stable isotopes and percent organic matter between each layer. Nutrient and isotopic results exhibited varying differences between microbial mat layers, with the majority of significant differences observed in the δ^15^N isotopic ratios (Figure 6). The percent of TOM was not found to be significantly different between different layers of the microbial mat; however, it was found to be significantly different between certain layers of the mat and the sand control (Kruskal–Wallis, *χ* ^2^ = 17.40, df = 6, *p* = 0.079), with Dunn's post hoc test revealing several pairwise differences. Specifically, the percent of total organic matter was significantly higher in Layers 2 and 3 compared to the sand control (Layer 2 vs. Sand: *z* = 3.16, *p* = 0.016; Layer 3 vs. Sand: *z* = 3.16, *p* = 0.033). Similarly, the percent of TOC was not found to be significantly different between layers of the microbial mat; however, it was significantly higher in Layer 2 compared to the sand control (Kruskal–Wallis, *χ* ^2^ = 15.96, df = 6, *p* = 0.014; Dunn's Test, Layer 2 vs. Sand: *z* = 3.36, *p* = 0.017). The percent of TN within the microbial mat samples followed a similar pattern. The percent of TN was not found to be significantly different between different layers of the microbial mat; however, it differed significantly between certain layers of the mat and the sand control (Kruskal–Wallis, *χ* ^2^ = 17.58, df = 6, *p* < 0.01), with Dunn's post hoc test revealing several pairwise differences. Specifically, the %TN was significantly higher in Layers 1 and 2 compared to the sand control (Layer 1 vs. Sand: *z* = 2.71, *p* = 0.048; Layer 2 vs. Sand: *z* = 3.14, *p* = 0.035).

There were no significant differences in δ^13^C isotopic ratios across microbial mat layers; however, δ^13^C was found to be significantly different between certain layers of the mat and the sand control (ANOVA: *F*_6,14_ = 3.564, *p* = 0.024). Following the ANOVA, Tukey's HSD post hoc test highlighted that Layer 4 of the microbial mat has a significantly higher δ^13^C value compared to the sand control (*p* = 0.031). When investigating the δ^15^N isotopic ratios, significant differences were found between microbial mat layers, as well as between several microbial mat layers and the sand control (ANOVA: *F*_6,14_ = 15.62, *p* < 0.001). Tukey's HSD post hoc test revealed that Layers 3, 4, 5, and 6 had significantly higher δ^15^N values than Layer 1 (*p* = 0.003, *p* < 0.001, *p* < 0.001, *p* < 0.001, respectively). Layers 5 and 6 were found to have significantly higher δ^15^N values compared to Layer 2 (*p* = 0.003 and *p* = 0.003, respectively). Additionally, the sand control had significantly higher δ^15^N values than Layer 1 (*p* = 0.020), and significantly lower values than Layers 5 and 6 (*p* = 0.021 and *p* = 0.021, respectively). Finally, to explore the relationship between the isotopic ratios of δ^13^C and δ^15^N in the microbial mats, raw data points were visually examined by plotting δ^13^C against δ^15^N, revealing sample groups by vertical layer (Figure 6).

## 4 Discussion

Mats exhibited vertically stratified layers with differences in diversity and community composition between the different strata (Figures 2 and 4). These results were consistent with other vertically stratified cyanobacterial mats in which small changes in depth showed distinct spatially separated niches and communities attributed to functional differences based on the specific abiotic and biotic conditions present in each layer, reflecting differences in available energy sources and associated metabolic pathways (Danovaro et al. 2024; Prieto‐Barajas et al. 2018). The overall functional role of coastal cyanobacterial mats in this system is evidenced by the strong pattern of differences in nutrient conditions between samples taken of bare sand and those taken of mat biomass (e.g., Figure 6), paired with differences in beta diversity between bare sand and mat biomass (Figure 3). Consistent with these findings, we found that mats were dominated by Proteobacteria, Bacteroidota, Planctomycetota, Desulfobacterota, Verrucomicrobiota, and Cyanobacteria, but with marked declines in Bacteroidota, Cyanobacteria, and Chlorflexi as anoxia develops with depth (Figure 4). Total organic matter, total organic carbon, and total nitrogen are highest in these upper layers, reflective of the high productivity and biomass of these phototrophs.

Members of Bacteroidota are common within marine salterns, seawater, sea sediment, and algal mats and may contribute to the regulation of soil nitrogen cycling (Pan et al. 2023). Within Bacteroidota, Flavobacteriaceae, Cylclobacteriaceae, Saprospiraceae, and Flammeovigaceae were the most abundant families. Many Bacteroidota are heterotrophic aerobes that undergo respiration, which is consistent with their higher abundance in the uppermost layers (Phelps et al. 1998; McIlroy and Nielsen 2014). Some members of the Flavobacteriaceae can be symbiotic with algae, while others within this family and in the Cylclobacteriaceae are algal predators (Pinnaka and Tanuku 2014; McBride 2014). As such, we do not expect those groups to have consistent carbon and nitrogen signatures. Cyanobacteria within the mats were dominated by the typical mat forming members found in supratidal and interdidal mats, *Coleofasciculus chthonoplastes*, *Lyngbya aestuarii* , both reported previously in North Carolina mats (Paerl et al. 1993; Paerl 1990). *Lyngbya* is capable of nitrogen fixation, consistent with higher total nitrogen content, and both of the dominant members form interwoven filaments with extracellular polymeric substances that can trap sediment and stabilize mats, consistent with increased total organic matter (Paerl et al. 1993; Bebout et al. 1987; Paerl 1990). As these are the main phototrophic bacteria within cyanobacterial mats, we expected to find these within the uppermost layers. Surprisingly, Chloroflexi decreased with depth as most members of this phyla are described as being anaerobic fermenters (Xia et al. 2016; Payne et al. 2024). The dominant order within our samples (Order SBR1031) was originally characterized from hot springs; however, recent metagenomic sequencing from stratified mats in the Turks and Caicos Islands has identified members that are considered to be facultative aerobes that can undergo aerobic respiration and phototrophy (Ward et al. 2020). These newly described functions from this order coincide with our observations of a higher abundance of Chloroflexi in the uppermost three layers.

Nutrient results support the above findings. In Layers 1 and 2, the total nitrogen may be attributed to zones of active microbial growth, and therefore, nitrogen accumulation near the mat surface, and potentially embedded within the extracellular polymeric substance of the microbial mats. This aligns with findings that ammonium, specifically, can become embedded in the upper 0.5 cm of Camargue mats from south France (Bonin and Michotey 2006), together suggesting that the upper mat matrix layers may act as a nitrogen reservoir. In Layers 2 and 3, we observed increases in total organic matter and total organic carbon (Figure 6). These strata likely represent zones of enriched primary production, where phototrophic organisms (such as Cyanobacteria and Proteobacteria mentioned above) fix CO_2_, contributing to elevated carbon pools. Because many microbes are phototrophic, their dependence on fixed carbon is reduced, allowing organic matter accumulation. At the same time, however, heterotrophic aerobes (such as Bacteroidota; Figure 4) may be present and interacting with the photoautotrophs; although they consume organic matter, in highly productive upper layers, organic inputs likely outpace consumption (Ataeian et al. 2022).

A transition zone at the overlap of aerobic and anaerobic processes approximately at Layers 3 and 4 is marked by a change in metabolism and δ^13^C (Figure 6). Heterotrophic bacteria inhabiting the transition zone between aerobic and anoxic regions produce carbon dioxide, acetate, hydrogen, and formate, which increase oxygen gradients downcore. This change in metabolism is likely linked to a peak in δ^13^C found in Layer 4, and a relative decline in total organic matter, including both total organic carbon and total nitrogen, between Layers 3 and 4 (Figure 6). In these transition layers, if oxygenic phototrophy (which tends to be more δ^13^C depleted) transitions to anoxygenic phototrophy or chemoautotrophy (less fractioning, more δ^13^C enriched), this signal may increase. This is supported by taxonomic data, as there was a high abundance of Proteobacteria found throughout all layers. The most common families within Proteobacteria were Alteromonadaceae, Halieaceae, Rhodobacteraceae, and Woeseiaceae. All of these families are common within marine sediments and can vary between aerobic and anaerobic lifestyles (Mußmann et al. 2017; Pohlner et al. 2019; Li et al. 2023). Alteromonadaceae, Halieaceae, and Rhodobacteraceae were most common in the uppermost layers, with Altermonadaceae being almost exclusively found in the first layer and Rhodobacteraceae, peaking in Layer 2, and then descreasing with depth. Halieaceae is known to be chemotrophic or photoheterotrophic with pigments that allow them to adapt to different depths as they can perform aerobic anoxygenic phototrophy (Li et al. 2023). Woeseiaceae was found to increase with depth and can degrade polysaccharides, assimilate and reduce nitrogen, and oxidize sulfur (Mußmann et al. 2017). This transition zone is highlighted by the shift from photosynthetic taxa to more heterotrophic and chemotrophic members further down with the layers.

Increasing δ^15^N with depth is indicative of increasing trophic level, likely related to the consumption of organic matter generated in the upper strata by heterotrophic bacteria in deeper, anoxic layers. This also suggests progressive N cycling, where there is more N processing, causing the heavier nitrogen to accumulate within these deeper, anoxic zones. Acidobacteriota, Desulfobacterota, and Planctomycetota were differentially expressed and found in a higher abundance in the deeper layers (Figure 4). Acidobacteriota is dominated by the family Thermoanaerobaculaceae within our samples, which is thought to be ubiquitous within soil and sediment samples and is more common when there is a higher contamination of nitrogen (Hamamoto et al. 2024; Dedysh and Yilmaz 2018). Thermoanaerobaculaceae and Desulfobacterota are both sulfur reducers and anaerobes (Fonseca et al. 2022). Classes that were differentially expressed within Desulfobacterota were Desulfobacteria and Desulfuromonadia, which are dominated by the genera SVA0081 Sediment Bacteria and Desulfuromusa, respectively. Both of these genera, which are common in marine sediments, are anaerobic and are chemoorganotrophic and can use some carbon sources, H_2_, and sulfur sources for energy (Fonseca et al. 2022; Liesack and Finster 2015). Additionally, Planctomycetota is also common in marine sediments, with Pirelluiaceae the most common family found in our samples. Pirelluiaceae was at higher abundance in lower layers and is known to be heterotrophic and capable of degrading numerous carbon and nitrogen sources (Wiegand et al. 2020). Species within this family have also been known to produce novel small molecules, including terpenes, bacteriocins, ecotiones, and antibiotics (Vitorino et al. 2022).

Our results do not indicate differences in community structure along a horizontal transect of the BCM (Figure 5), contrasting with other studies (Huang et al. 2020; Farias et al. 2017; Bolhuis and Stal 2011; Cadena et al. 2019). However, other work on BCMs that found horizontal gradients were often conducted at larger scales focusing on differences between sites or other environmental parameters and were not conducted within a single contiguous BCM. For example, in microbial mats from North Carolina and from Massachusetts, USA, differences in bacterial members were based on mat maturity, where more recently established mats exhibited less stratification than more mature mats (Paerl et al. 1993; Armitage et al. 2012). We did not find a similar pattern within our samples, as cores taken near the center of the mats, which might be classified as more mature, did not differ from those taken near the edge, which could be classified as newer, developing mat areas. BCMs at our sites were roughly continuous, covering the majority of exposed sand. Additional exploration of BCM communities during mat growth and senescence phases through controlled manipulations may provide a more direct test of community maturity at these sites in the future.

## 5 Conclusion

This work represents the first molecular characterization of the full bacterial community from BCMs from North Carolina and provides insights into the potential biogeochemical processes within this system. Building off of previous studies (Paerl et al. 1993, 1991; Bebout et al. 1987; Bautista and Paerl 1985), we found the same cyanobacterial members as previously described and characterized additional phyla, with the dominant members in Proteobacteria, Bacteroidota, Planctomycetota, and Desulfobacterota. Community composition and species richness differed between vertical strata of these BCMs but did not reveal horizontal structure. Biological stratification within the mat was associated with nutrient and isotopic gradients that varied with depth and associated microbial functions. Because we focused on a fine‐scale resolution of the composition of bacterial communities, additional work is needed to characterize specific strains and the potential function of the bacterial community within these BCMs through metagenomic and metatranscriptomic analyses. Additionally, to understand further how BCM community composition and function vary over space, this study could be expanded to characterize the variation more broadly by investigating mat maturity and variation across sites, seasons, and abiotic metrics.

## Author Contributions

**Schyler A. Ellsworth:** conceptualization (lead), data curation (equal), formal analysis (equal), project administration (equal), visualization (equal), writing – original draft (equal), writing – review and editing (equal). **Madelina S. Marquez:** conceptualization (equal), funding acquisition (equal), visualization (equal), writing – original draft (equal), writing – review and editing (equal). **Sophie J. McCoy:** conceptualization (equal), funding acquisition (lead), project administration (equal), supervision (equal), writing – original draft (equal), writing – review and editing (equal).

## Funding

This work was supported by the Phycological Society of America, Grant‐in‐Aid of Research Award; Division of Ocean Sciences (2023357209, OCE‐2239425); and College of Arts and Sciences, University of North Carolina, Royster Society Fellowship, Start‐up Funding, Wilson Grant by UNC Biology Department.

## Conflicts of Interest

The authors declare no conflicts of interest.

## Data Availability

Raw sequencing reads for data generated for this project were submitted to the National Center for Biotechnology Information's (NCBI) Sequence Read Archive (SRA) under the BioProject PRJNA1337146 and BioSample accession numbers SAMN52169421–SAMN52169457. Code used for data analysis is available on https://github.com/schyells/DNA_Metabarcoding_Analysis.
